# Modelling hydrogen production from biomass pyrolysis for energy systems using machine learning techniques

**DOI:** 10.1007/s11356-023-27805-5

**Published:** 2023-05-30

**Authors:** Paulino José García-Nieto, Esperanza García-Gonzalo, Beatriz María Paredes-Sánchez, José Pablo Paredes-Sánchez

**Affiliations:** 1 Department of Mathematics, Faculty of Sciences, University of Oviedo, 33007 Oviedo, Spain; 2 Department of Energy, College of Mining, Energy and Materials Engineering, University of Oviedo, 33004 Oviedo, Spain

**Keywords:** Bioenergy, Hydrogen gas production (HGP), Multilayer perceptron (MLP), Support vector regression (SVR), Artificial bee colony (ABC)

## Abstract

In the context of Industry 4.0, hydrogen gas is becoming more significant to energy feedstocks in the world. The current work researches a novel artificial smart model for characterising hydrogen gas production (HGP) from biomass composition and the pyrolysis process based on an intriguing approach that uses support vector machines (SVMs) in conjunction with the artificial bee colony (ABC) optimiser. The main results are the significance of each physico-chemical parameter on the hydrogen gas production through innovative modelling and the foretelling of the HGP. Additionally, when this novel technique was employed on the observed dataset, a coefficient of determination and correlation coefficient equal to 0.9464 and 0.9751 were reached for the HGP estimate, respectively. The correspondence between observed data and the ABC/SVM-relied approximation showed the suitable effectiveness of this procedure.

## Introduction

Industry 4.0 is defined as the fourth industrial revolution, which refers to the ongoing automation and digitisation of industrial processes and systems, incorporating technologies such as the Internet of Things (IoT), artificial intelligence (AI) (e.g. machine learning), cloud computing, and robotics. As Industry 4.0 moves towards a cleaner environment, it advocates for increased use of sustainable energy and parametric characterisation of energy. To ensure improved energy resources, the world faces the challenge of promoting the use of renewable energy sources (Adekoya et al. 2021) and reducing carbon emissions (Qadir et al. 2021).

Bioenergy management can help reduce a portion of greenhouse gas (GHG) emissions and achieve sustainable goals in energy production systems (Paredes-Sánchez et al. 2020). Biomass pyrolysis is considered a promising technology for producing hydrogen because it allows for the direct conversion of energy resources from a solid to a gaseous state. Hydrogen is widely used as a primary energy carrier in sustainable energy applications. It can be obtained from both non-renewable sources, such as natural gas, and renewable resources, such as biomass, for energy conversion and management (Abe et al. 2019). Combusting hydrogen in energy systems is highly environmentally friendly since only water vapour is produced (Ni et al. 2006). Hydrogen has significant energy storage capacity, with an energy density of around 120 MJ/kg, more than double that of most conventional fuels (Sherif et al. 2014).

**Fig. 1:**
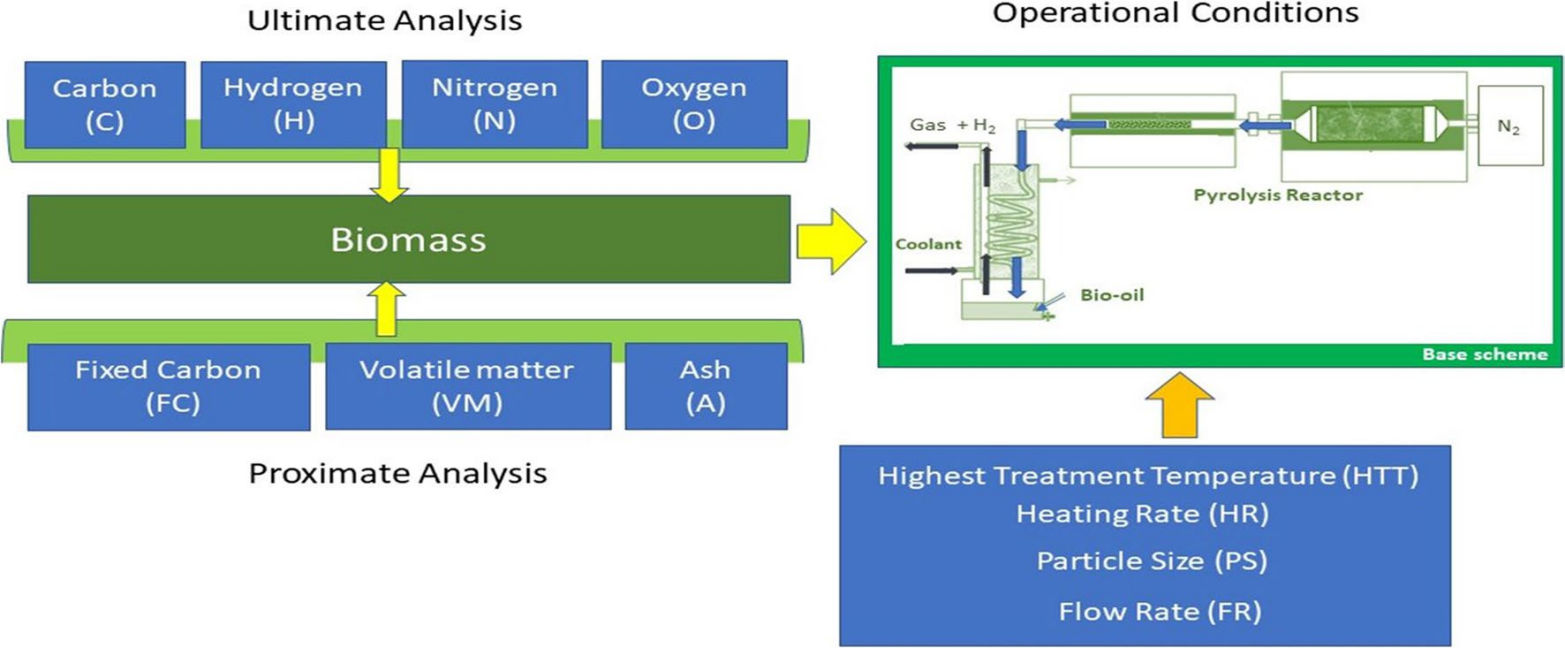
Scheme of the experimental procedure

Pyrolysis involves heating organic material at or above 500 °C in the absence of oxygen, using inert gas under controlled operating conditions, including inert gas flow rate (FR), heating rate (HR), particle size (PS), and highest treatment temperature (HTT). This process produces combustible gases (Ahrenfeldt 2012) and biochar (Rosillo-Calle and Woods 2012) by thermally decomposing biomass. However, the intrinsic characteristics of solid biomass, such as high moisture content, hydrophilic nature, poor heating value, and low bulk density, make it necessary to characterise biomass parameters to determine its potential conversion to hydrogen by pyrolysis. Proximate analysis parameters, such as volatile material (VM), fixed carbon (FC), and ash (A), as well as ultimate analysis parameters, including carbon (C), hydrogen (H), nitrogen (N), and oxygen (O), are used to structure models to select raw materials more suitable for hydrogen production by pyrolysis. The basic equipment for biomass composition analysis includes a pyrolysis reactor with necessary additional automatic equipment for biomass composition analysis, such as thermobalances or ovens, to characterise proximate and ultimate analysis parameters (Ahrenfeldt 2012; García-Nieto et al. 2019) or a combination of both analyses (Yin 2011) (see Fig. 1).

To improve the experimental process, there is a rising need to forecast the hydrogen gas production (HGP) of biomass pyrolysis utilising data from proximal and ultimate studies employing algorithms relied on statistical machine learning techniques. Machine learning is a part of artificial intelligence and computer science whose aim is to build strategies that permit computers to acquire knowledge. Computers observe data, build models based on that data by machine learning techniques, using the model as a hypothesis about the analysed process, and implement it in software to find solutions to the problem. In the references, some research studies show the use of machine learning techniques for similar problems. In this sense, the previous study to evaluate the gasification of biomass for the production of hydrogen, and its modelling by means of supervised machine learning algorithms, is noteworthy (Ozbas et al. 2019). The analysis of pyrolytic gas yield and compositions with feature reduction methods by the machine learning prediction, considering the effects of biomass characteristics and pyrolysis conditions, is an interesting study carried out in the same research way (Tang et al. 2021). In this regard, the research work that employs mathematical techniques relied on the machine learning for modelling the gasification and pyrolysis of biomass and waste is also noteworthy (Ascher et al. 2022).

When the raw biomass is used in the pyrolysis process to feed into a reactor, it heats up. As the temperature increases, the particles eject product gases (e.g. H_2_). Product gases are mixed up with the flowing inert gas and are conducted to the reactor outlet, after a cooling step to avoid product degradation (Lathouwers and Bellan 2001). In these conditions, hydrogen is produced together with other product gases.

The main reason for the present research is to implement support vector machines (SVMs) (Cristianini and Shawe-Taylor 2000; James et al. 2021) in conjunction with the metaheuristic optimiser termed as artificial bee colony (ABC) (Eberhart et al. 2001; Chong and Zak 2013; Aggarwal 2020) and the multilayer perceptron (MLP) (Du and Swamy 2019; Kubat 2021) as well as M5 model tree (M5Tree) (Singh et al. 2016) to evaluate the hydrogen gas production (HGP) in the pyrolysis procedure from biomass for bioenergy. Based on measurable learning hypotheses, the SVM approach is another category of strategies that can be employed to foretell values ​​from very distinct domains (Hansen and Wang 2005; Steinwart and Christmann 2008).

The ABC optimiser has been implemented here with successful results to carry out the optimisation stage for the purpose of determining the optimal kernel hyperparameters during the SVM training. ABC is basically an analysation algorithm that allows a selection of the most optimal values for a problem optimisation from a metaheuristic point of view.

Accordingly, a novel approach that combines the ABC optimiser and SVM-relied regressor was evaluated as automatic apprenticeship algorithms, training them to estimate the HGP based on raw biomass characteristics (e.g. proximate analysis, ultimate analysis) and pyrolysis conditions. In this sense, investigators have effectually employed the SVM method in research works of energy matters like photovoltaic energy (de Leone et al. 2015), solar radiation (Chen et al. 2013), or hydro-climatic factors (Shrestla and Shukla 2015). Additionally, a multilayer perceptron-like neural network (MLP approach) and M5 model tree were fitted to the experimental biomass dataset for comparison objectives (Kisi 2015; Du and Swamy 2019; García-Nieto et al. 2019). MLP is a type of artificial neural network (ANN) formed by multiple layers, in such a way that it has the capacity to find solutions to problems that are not linearly separable. Furthermore, the M5 model tree is a learning decision tree that performs regression work, which means that it is employed to foretell the values of an output numerical attribute. The approach based on M5 employs the mean square error as an impurity function analogously to the approximation based on the CART tree (Kuhn and Johnson 2018). The principal difference between CART tree and M5 model tree is that the first one assigns a constant to the leaf node, while the second one fits a multivariate linear regression function. Therefore, the M5 model tree consists of piecewise linear functions. Another additional benefit of the M5 model tree is that it can acquire knowledge effectively and can deal with works with a very elevated dimensionality (Kisi 2015): up to hundreds of attributes.

Therefore, a SVM-relied approximation optimised with ABC (ABC/SVM) together with MLP-relied ANN and M5 model tree was utilised as a statistical learning-relied tool for the purpose of training the data in order to estimate the HGP from the attributes of the proximate analysis, ultimate analysis, and operational conditions of the pyrolysis process.

## Materials and methods

There are plenty of mathematical approaches, which were constructed based on experimental data from raw biomass and pyrolysis process to define energy conversion (Lee et al. 2021).

### Materials

The main base of the employed dataset for this investigation is a group of experimental proximate and ultimate analyses and the conditions of the pyrolysis process with their corresponding hydrogen gas productions (HGPs) (dependent variable) based on the product yields (Demiral and Ayan 2011) and main product characteristics (Beis et al. 2002; Bordoloi et al. 2016) from the pyrolysis process. The total data processed was about 1272 values (106 data per variable × 12 variables (11 independent variables and 1 dependent variable)) (Han et al. 2011; Frank et al. 2016). These values were acquired from the proximate and ultimate analyses of biomass, and the next pyrolysis process for hydrogen production obtained in earlier studies. Additionally, to make decisions in the energy conversion systems and energy carriers, decision-makers and investigators require analysis models (Garg et al. 2016) to assess advanced energy efficiency (Paredes-Sánchez et al. 2019).

Concerning the input variables, the main ones have been obtained from the ultimate and proximate analyses of biomass, and taking into account the pyrolysis conditions for an operational evaluation of hydrogen gas production (HGP) (dependent variable). The dataset treated with the three kinds of approximations (ABC/SVM-relied approach, MLP-relied approximation, and M5 model tree) relies on some operational production parameters and various physico-chemical attributes of the pyrolysis process (Morali and Şensöz 2015; Safdari et al. 2018).

The physical–chemical input variables and pyrolysis production parameters of the model are outlined below (Yuan et al. 2015):

- Proximate analysis:

- Ash refers to the inorganic matter impurities that remain after the combustion of the biomass.
- Fixed carbon (FC) refers to the inorganic matter impurities that remain after the combustion of the biomass.
- Volatile matter (VM): Measured by determining the loss of weight of the raw material.

- Ultimate analysis (Encinar et al. 2000; Gong et al. 2020):

- Carbon (C): The carbon fraction in the elemental composition of the raw biomass.
- Hydrogen (H): The hydrogen content determined by elemental analysis of the raw biomass. It has a direct relationship with hydrogen production in pyrolysis gases.
- Nitrogen (N): The nitrogen fraction in the elemental composition of the raw biomass.
- Oxygen (O): The oxygen content of the raw biomass determined by elemental analysis.

- Operational production conditions of the pyrolysis process:

- Highest treatment temperature (HTT): The highest temperature reached during the pyrolysis process for hydrogen production.
- Heating rate (HR): The rate of heat applied to convert the raw biomass into hydrogen through pyrolysis.
- Particle size (PS): The size of the particles used in the chemical reactor during the pyrolysis process.
- Flow rate (FR): The rate at which inert gas is introduced into the pyrolysis reactor.

### Methods

#### Support vector regression (SVR) method

In this subsection, the use of support vector machines (SVM) to find solutions to regression problems is studied. In these cases, they are usually called support vector regression (SVR) (Cristianini and Shawe-Taylor 2000). Consider a collection of training examples $+\left\{\left({\mathbf{x}}_{1},{y}_{1}\right),\dots ,\left({\mathbf{x}}_{n},{y}_{n}\right)\right\}$, where ${\mathbf{x}}_{i}\in {\mathfrak{R}}^{d}$ and ${y}_{i}\in \mathfrak{R}$, assuming that the *y*_*i*_ values of all examples of *S* can be fitted (or quasi-fitted) by a hyperplane; our goal is to find the parameters $\mathbf{w}=\left({w}_{1},\dots ,{w}_{d}\right)$ that allow us to define the regression hyperplane $f\left(\mathbf{x}\right)=\left({w}_{1}{x}_{1}+{w}_{2}{x}_{2}+\dots +{w}_{d}{x}_{d}\right)+b=\langle \mathbf{w},\mathbf{x}\rangle +b$.

The random noise or disturbance $\varepsilon \sim N\left(0,\sigma \right)$ is defined as the measurement’s error of the value *y*, that is, $y=f\left(\mathbf{x}\right)+\varepsilon$. To allow for some noise in the training examples, we can relax the error condition between the value foretold by this function and the real value. For this, the $\varepsilon -$ insensitive loss function is used, ${L}_{\varepsilon }$, given by (James et al. 2021)

1 $$L_\varepsilon\left(\mathbf x\right)=\begin{Bmatrix}0&\begin{array}{cc}\mathrm{if}&\left|y-f(\mathbf x)\right|\leq\varepsilon\end{array}\\\left|y-f\left(\mathbf x\right)\right|-\varepsilon&\mathrm{otherwise}\end{Bmatrix}$$

It is a linear function with an insensitive zone of width $2\varepsilon$, in which the loss function takes a null value. By choosing this function, some flexibility in the solution function is allowed, so that all the examples that are confined to the tubular region will not be considered support vectors, since the cost associated with the loss function is 0. In practice, it is very difficult to achieve a linear regression model with zero prediction error, so the concept of *soft margin* will be introduced (Sugiyama 2015).

**Fig. 2:**
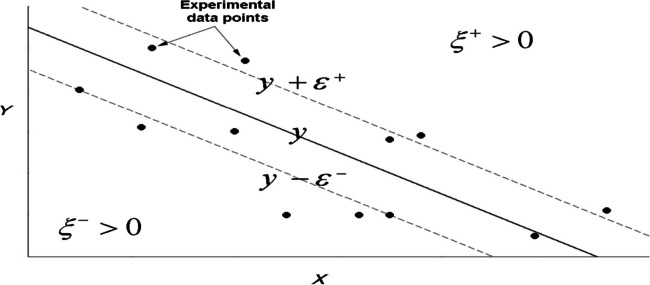
An illustration of the $\varepsilon -$ insensitive tube in the event of regression

The slack variables are defined as the distance to the sample measured from the tubular zone of the regression hyperplane. The slack variables ${\xi }_{i}^{+}$ and ${\xi }_{i}^{-}$ will allow us to quantify the prediction error that is willing to admit for each training example and, with the sum of all of them, the cost associated with the examples with a non-zero prediction error. ${\xi }_{i}^{+}>0$ will be taken when the prediction of the example $f\left({\mathbf{x}}_{i}\right)$ is greater than its actual value, ${y}_{i}$, in an amount greater than $\varepsilon$ or equivalently $f\left({\mathbf{x}}_{i}\right)-{y}_{i}>\varepsilon$. Similarly ${\xi }_{i}^{-}>0$ when the actual value of the example is greater than its prediction by an amount greater than $\varepsilon$, that is, ${y}_{i}-f\left({\mathbf{x}}_{i}\right)>\varepsilon$. In any other case, the slack variables take a value of 0. Note that both variables cannot simultaneously take a value other than 0, since it happens whenever ${\xi }_{i}^{+}\cdot {\xi }_{i}^{-}=0$ (see Fig. 2).

With all this, now the problem can be optimised. Our goal is to minimise the sum of the associated loss functions, each one to an example of the training set $\sum_{i=1}^{n}{L}_{\varepsilon }\left({y}_{i},f\left({\mathbf{x}}_{i}\right)\right)={\sum }_{i\in \mathrm{non}-\mathrm{tubular\;zone}}\left|{y}_{i}-f\left({\mathbf{x}}_{i}\right)\right|-\varepsilon$. This is equivalent to maximising the tubular zone defined by the loss function, in which it takes a null value. Therefore, maximising $\varepsilon$ is equivalent to minimising $\Vert \mathbf{w}\Vert$. All these together with the penalty imposed by the slack variables defines the following optimisation problem with soft margin, so that *C* is called the *regularisation constant* (Li et al. 2008):

2 $$\begin{array}{l}\displaystyle\min_{w,b,\xi^+,\xi^-}\frac12{\Arrowvert\mathbf w\Arrowvert}^2+C\sum\limits_{i=1}^n\left(\xi_i^++\xi_i^-\right)\\\mathrm{subject}\;\mathrm{to}\\\begin{Bmatrix}\left(\langle\mathbf w,{\mathbf x}_i\rangle+b\right)-y_i-\varepsilon-\xi_i^+\leq0&i=1,\dots,n\\y_i-\left(\langle\mathbf w,{\mathbf x}_i\rangle+b\right)-\varepsilon-\xi_i^-\leq0&i=1,\dots,n\\\xi_i^+,\xi_i^-\geq0&i=1,\dots,n\end{Bmatrix}\end{array}$$

Next, the transformation to the dual problem with four families of Lagrange multipliers (${\alpha }_{i}^{+},{\alpha }_{i}^{-},{\beta }_{i}^{+},{\beta }_{i}^{-}$) is carried out (Steinwart and Christmann 2008):

3 $$\begin{array}{l}\begin{array}{c}\max\\\varvec\alpha^+,\varvec\alpha^-\end{array}\sum\limits_{i=1}^n\left(\alpha_i^--\alpha_i^+\right)y_i-\varepsilon\sum\limits_{i=1}^n\left(\alpha_i^-+\alpha_i^+\right)-\frac12\sum\limits_{i,j=1}^n\left(\alpha_i^--\alpha_i^+\right)\left(\alpha_j^--\alpha_j^+\right)\langle{\mathbf x}_i,{\mathbf{x}}_{i}\rangle\\\mathrm{subject}\;\mathrm{to}\\\begin{Bmatrix}\begin{array}{cc}\sum\limits_{i=1}^n\left(\alpha_i^+-\alpha_i^-\right)=0&\mathrm{with}\end{array}\\\begin{array}{cc}0\leq\alpha_i^+,\alpha_i^-\leq C&i=1,\dots,n\end{array}\end{Bmatrix}\end{array}$$

The obtained regressor is (Pal and Goel 2007)

4 $$f\left(\mathbf{x}\right)=\sum_{i=1}^{n}\left({\alpha }_{i}^{-}-{\alpha }_{i}^{+}\right)\langle \mathbf{x},{\mathbf{x}}_{i}\rangle +{b}^{*}$$

The optimal value ${b}^{*}$ is obtained from the restrictions resulting from the application of the second *Karush–Kuhn–Tucker* (KKT) condition and the restrictions on the dual problem, so that (Zeng and Qiao 2013)

5 $$\left\{\begin{array}{c}\begin{array}{ccc}{b}^{*}={y}_{i}-\langle {\mathbf{w}}^{*},{\mathbf{x}}_{i}\rangle +\varepsilon & \mathrm{if}& 0<{\alpha }_{i}^{+}<C\end{array}\\ \begin{array}{ccc}{b}^{*}={y}_{i}-\langle {\mathbf{w}}^{*},{\mathbf{x}}_{i}\rangle -\varepsilon & \mathrm{if}& 0<{\alpha }_{i}^{-}<C\end{array}\end{array}\right\}$$

Note that to define the regression hyperplane, the examples with a non-zero loss function are considered, that is, those that are outside the tubular region. Viewed in terms of the parameters introduced above, for the support vectors it gathers from the Karush–Kuhn–Tucker (KKT) conditions that ${\alpha }_{i}^{+}\cdot {\alpha }_{i}^{-}=0$, so (Hansen and Wang 2005).

- for the examples that are outside the tubular zone, it will be fulfilled ${\xi }_{i}^{+}\cdot {\xi }_{i}^{-}=0$, if ${\xi }_{i}^{-}=0$ and ${\xi }_{i}^{+}>0$, then ${\alpha }_{i}^{+}=C$ and ${\alpha }_{i}^{-}=0$; and if ${\xi }_{i}^{-}>0$ and ${\xi }_{i}^{+}=0$, then ${\alpha }_{i}^{-}=C$ and ${\alpha }_{i}^{+}=0$;
- the support vectors that are just into the border of the sensitivity zone verify that if $0<{\alpha }_{i}^{+}<C$, then ${\alpha }_{i}^{-}=0$. In that case, it must be ${\xi }_{i}^{+}=0$ and ${\xi }_{i}^{-}=0$. Similarly for the other case.

The examples which ${\alpha }_{i}^{+}={\alpha }_{i}^{-}=0$ (are not considered support vectors) are found within the tubular region.

When the examples cannot be fitted by a linear function (nonlinear problems), the use of *kernel functions* is mandatory. Through a suitable kernel, a Hilbert space is induced, also called a feature space, in this it is possible to adjust the transformed examples using a linear regressor, which has the following expression (García-Nieto et al. 2020):

6 $$f\left(\mathbf{x}\right)=\sum_{i=1}^{n}\left({\alpha }_{i}^{-}-{\alpha }_{i}^{+}\right)K\left(\mathbf{x},{\mathbf{x}}_{i}\right)$$

Now the coefficients are obtained solving the dual problem that results from Eq. (3) with dot products substituted for kernel functions (Nikoo and Mahjouri 2013).

7 $$\begin{array}{l}\begin{array}{c}\max\\\varvec{\alpha}^+,\varvec{\alpha}^-\end{array}\sum\limits_{i=1}^n\left(\alpha_i^--\alpha_i^+\right)y_i-\varepsilon\sum\limits_{i=1}^n\left(\alpha_i^-+\alpha_i^+\right)-\frac12\sum\limits_{i,j=1}^n\left(\alpha_i^--\alpha_i^+\right)\left(\alpha_j^--\alpha_j^+\right)K\left({\mathbf x}_i,{\mathbf x}_j\right)\\\mathrm s\mathrm u\mathrm b\mathrm j\mathrm e\mathrm c\mathrm t\;\mathrm t\mathrm o\\\begin{Bmatrix}\sum\limits_{i=1}^n\left(\alpha_i^+-\alpha_i^-\right)=0&\mathrm{with}\\0\leq\alpha_i^+,\alpha_i^-\leq C&i=1,\dots,n\end{Bmatrix}\end{array}$$

To solve regression problems using SVRs, a suitable kernel and a *C* parameter must be chosen as well as the selection of a suitable $\varepsilon$. The value of the parameter *C* expresses the balance between the flatness of the objective function and the decrease of the model complexity (Schölkopf et al. 2000). In the case of noisy regression problems, the parameter $\varepsilon$ should be selected to express the variance of the noise in the data, since in most practical cases, it is possible to obtain an approximate measure of the noise variance from the training data. The methodology employed to choose the optimal values of *C* and the rest of the kernel parameters is normally based on cross-validation techniques (Chen et al. 2022).

Several frequent functions used as kernels in the research publications are given by the following (Ziani et al. 2017):

- Polynomial kernel:
  8 $$K\left({\mathbf{x}}_{i},{\mathbf{x}}_{j}\right)={\left({\sigma \mathbf{x}}_{i}\cdot {\mathbf{x}}_{j}+\alpha \right)}^{b}$$
- Sigmoid kernel:
  9 $$K\left({\mathbf{x}}_{i},{\mathbf{x}}_{j}\right)=\mathrm{tanh}({\sigma \mathbf{x}}_{i}\cdot {\mathbf{x}}_{j}+a)$$
- RBF (radial basis function) kernel:
  10 $$K\left({\mathbf{x}}_{i},{\mathbf{x}}_{j}\right)={e}^{-\sigma {\Vert {\mathbf{x}}_{i}-{\mathbf{x}}_{j}\Vert }^{2}}$$

where *a*, *b*, and $\sigma$ are the kernel hyperparameters.

Hence, to find the solution of a complicated regression problem like this, the SVM technique with data that is not linearly separable is used here. To this end, it is mandatory to select a kernel type along with its optimal parameters so that these data become linearly separable in a higher dimensional space (or *feature space*) (Ortiz-García et al. 2010).

#### Artificial neural network: multilayer perceptron (MLP)

Minsky and Papert showed in 1969 that the simple perceptron and ADALINE (adaptative linear element) cannot solve nonlinear problems (for example, XOR). The combination of several simple perceptrons could solve certain nonlinear problems, but there was no automatic mechanism to adapt the weights of the hidden layer. Rumelhart and other authors, in 1986, presented the *generalised delta rule* (GDL) to adapt the weights by propagating the errors backwards, that is, propagating the errors towards the lower hidden layers (Aggarwal 2018). In this way, it is possible to work with multiple layers and with nonlinear activation functions. It can be shown that this multilayer perceptron (MLP) is a universal approximator. A multilayer perceptron can approximate nonlinear relationships present between input and output data. This ANN has become one of the most common architectures.

The MLP is an artificial neural network (ANN) made up of multiple layers, in such a way that it can find solutions to problems that are not linearly separable. This matter is the principal limitation of the simple perceptron. However, MLP can be fully or locally connected. To be fully connected, all the neurons of a layer must be connected with all the neurons of the next layer, while this condition is not present in a locally connected MPL.

**Fig. 3:**
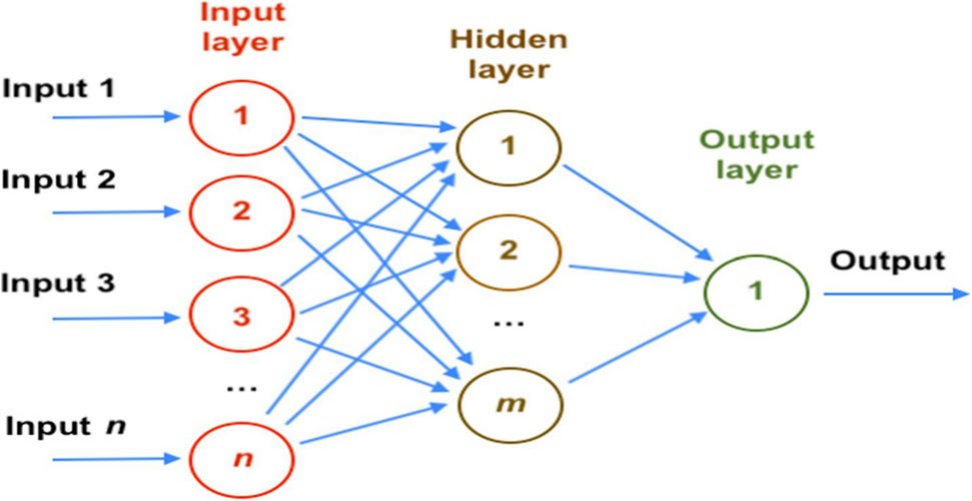
Artificial neural network of multilayer perceptron type with *n* input neurons, one output neuron, and its hidden layer made up of *m* neurons

The layers of an MLP can be classified into three types (see Fig. 3) (Du and Swamy 2019).

- Input layer: the information of the independent variables enters through this layer and there is no process here.
- Output layer: the connection with the dependent variables is made here.
- Hidden layers are layers located between the input and output layers that pass and process the information from the input to the output layers.

Backpropagation (also known as error backpropagation or *generalised delta rule*) is the mathematical rule to train this type of neural networks. In this sense, MLP is also termed as a backpropagation artificial neural network (BP-ANN). Additionally, the main quality of this kind of networks is that the transfer functions of the processing elements (neurons) must be *derivable*.

Learning occurs in the multilayer perceptron (MLP) by changing the weights of the connections considering the difference between the expected and the obtained output values. This change is performed using backpropagation which is a generalisation of the lowest mean square (LMS) used on the linear perceptron. For data point *n* the error at node *j* is ${e}_{j}\left(n\right)={d}_{j}(n)-{y}_{i}(n)$, being *d* the observed value and *y* the value predicted by the multilayer perceptron. The total error to correct is (Aggarwal 2018)

11 $$\varepsilon \left(n\right)=\frac{1}{2}\sum_{j}{e}_{j}^{2}(n)$$

Using the *gradient descent method*, the change of the weights is given by (Kuhn and Johnson 2018)

12 $${\Delta w}_{ji}\left(n\right)=-\eta \frac{\partial \varepsilon (n)}{{\partial v}_{j}(n)}{y}_{i}(n)$$

where

- $\eta$ is the *learning rate.* It must be chosen carefully: a small value produces a slow convergence, while a big value can hamper the convergence of the optimisation. Adequate values range from 0.2 to 0.8; and
- ${y}_{i}$ is the output obtained from the neuron in the previous layer.
- ${v}_{j}$ is the local induced field. It can be proved that for a given output node,
  13 $$-\frac{\partial \varepsilon (n)}{{\partial v}_{j}(n)}={e}_{j}(n)\cdot {\phi }^{^{\prime}}\left({v}_{j}\left(n\right)\right)$$

being ${\phi }^{^{\prime}}$ the derivative of the activation function. The variation of the weights for the nodes of the hidden layer is given by (Aggarwal 2018)

14 $$-\frac{\partial \varepsilon (n)}{{\partial v}_{j}(n)}={\phi }^{^{\prime}}\left({v}_{j}\left(n\right)\right)\sum_{k}-\frac{\partial \varepsilon (n)}{{\partial v}_{k}(n)}{w}_{kj}(n)$$

*k* is the subscript of the nodes from the output layer and these nodes affect the change of the weights of the hidden layer. It starts by changing the weights of the output layer taking into account the derivative of the activation function and then this process backpropagates modifying the weights of the previous layers.

#### Artificial bee colony (ABC) algorithm

This technique was first proposed by Karaboga (Karaboga 2005; Simon 2013) and is relied on the foraging behaviour of honey bees. Therefore, it belongs to the type of algorithms with a behaviour based on the exchange of information between entities that form a group (Karaboga and Basturk 2007; Karaboga and Akay 2009). It is a flexible algorithm able to solve real-world problems where an optimisation process is required. Although the initial applications of ABC were in numerical optimisation, current research topics extend ABC to the optimisation of hybrid functions, engineering design problems, multi-objective optimisation problems, neural network training, and image processing problems, among others.

An ABC swarm is a set of bees able to collaborate and communicate among themselves to perform the task of collecting. ABC uses bees with three different roles: (a) employed; (b) onlooker; and (c) scout bees (Tereshko and Loengarov 2005; Karaboga et al. 2014). Employed bees are related to the source of food. They share information such as distance and direction from the hive with the onlooker bees (Tereshko and Loengarov 2005). They select sources of food using the knowledge shared by employed bees. Onlooker bees choose high-quality food sources with more probability than low-quality food sources. The scouts search for new sources of food around the hive (Blum et al. 2008).

In ABC, the hive is made up of the same number of the onlooker and employed bees. The swarm’s food sources, or tentative solutions, depend on the number of onlooker and employed bees. Initially, ABC creates a random population of SN food sources or solutions. Given the food source (Karaboga et al. 2014),

15 $${X}_{i}=\left\{{x}_{i1},{x}_{i2},\dots ,{x}_{in}\right\}$$

a ${V}_{i}$ is generated by every employed bee in the proximity of its position following the equation (Tereshko and Loengarov 2005; Karaboga et al. 2014)

16 $${V}_{ik}={X}_{ik}+{\Phi }_{ik}\cdot \left({X}_{ik}-{X}_{jk}\right)$$

where ${X}_{j}$ is a solution $\left(i\ne j\right)$ that is selected randomly, *k* is an index randomly selected from the set $\left\{\mathrm{1,2},\dots ,n\right\}$ that indicates the chosen dimension, and ${\Phi }_{ik}$ is a random number in $\left[-\mathrm{1,1}\right]$. Once the ${V}_{i}$ candidate solution is obtained, if the new value ${V}_{i}$ improves that of its father ${X}_{i}$, then ${X}_{i}$ is updated with ${V}_{i}$. Otherwise, the value of ${X}_{i}$ is kept unchanged. Once the search process ends, the employed bees give the position of their food sources to the onlooker bees through their dances. Then, the onlooker bee assesses the information on the collected nectar and picks up a food source, taking into account a probability that is related to the amount of nectar in it. The probabilistic selection constitutes a mechanism of selection of roulette which is described in the following equation (Blum et al. 2008):

17 $${P}_{i}=\frac{{fit}_{i}}{\sum\limits_{j}{fit}_{j}}$$

where the fitness for a food source *i* is ${fit}_{i}$. Therefore, the better the food source *i*, the higher the probability it remains in the set of tentative solutions. If one of the food sources does not increase its fitness after some iterations, it is discarded. The scout bee is responsible for finding a replacement for the discarded source, following the equation (Karaboga et al. 2014)

18 $${X}_{ik}={lb}_{j}+ran\left(\mathrm{0,1}\right)\times \left({ub}_{j}-{lb}_{j}\right)$$

where $\mathrm{ran}\left(\mathrm{0,1}\right)$ is a random number from $\left[\mathrm{0,1}\right]$ relied on a uniform distribution being *lb* and *ub* the lower and upper boundaries that correspond to the *i*-th dimension, respectively.

#### M5 model tree

This machine learning technique has employed the following idea (Kisi 2015; Kubat 2021): the parameter space is split up into subspaces and then a linear regression model is constructed in each of them. The consequential model would be considered a modular model, in which the linear models specialise in the specific subsets of the input space.

The mathematical technique termed algorithm M5 is employed to force a model tree (James et al. 2021; Kisi 2015). Indeed, a group of *T* training data is considered here. Each instance is depicted by the values of a not variable collection of input attributes as well as a related goal output value. The principal goal is to build a method that connects an objective value of the training data with their input attribute values. The model excellence will usually be assessed if it foretells the objective values of the unknown cases accurately.

The method used to build tree-based machine learning models is *divide-and-conquer* (Rahimikhoob et al. 2013; Singh et al. 2016). The set *T* is linked to a leaf or several tests are selected to divide *T* into subsets. This splitting algorithm is applied recursively. The division criterion used by the M5 model tree algorithm makes use of the value of the standard deviation of the class values arriving at a node as a measure of the error at that node and then the calculation of the expected reduction of this error to check every attribute in that node. Indeed, the standard deviation reduction (SDR) can be determined by using the following expression (Pal and Deswal 2009; Behnood et al. 2015):

19 $$SDR=sd\left(T\right)-\sum \frac{\left|{T}_{i}\right|}{\left|T\right|}sd\left({T}_{i}\right)$$

where *T* indicates the number of examples arriving at the node, ${T}_{i}$ signifies the subset of cases that have the *i*th outcome of the potential collection, and *sd* is the standard deviation (Rahimikhoob et al. 2013; Seghier et al. 2018).

After a thorough examination of all potential splits, the M5 model tree selects the element that fully improves the expected error reduction (Pal 2006). This M5 model tree splitting mechanism ends when the class values of all instances arriving at a node differ by only a very small tolerance (stopping criterion), or else when only a few instances remain. This persistent splitting process often gives place to much elaborated structures that must be pruned, i.e. substituting a subtree by a leaf. With time, it is necessary to carry out a smoothing process to counterbalance for the abrupt discontinuities that will inevitably happen among adjacent linear models at the leaves of the pruned tree, in particular for several models built from a lower number of training data. During this procedure, the adjacent linear equations are upgraded so that the foretold outputs for the contiguous input vectors related to the distinct equations are transformed very close in their expressions (Khorrami et al. 2020).

#### Goodness of fit

The main goodness-of-fit statistics for the regression problem posed in this paper is the coefficient of determination *R*^2^ (Agresti and Kateri 2021). If the experimental and predicted values are ${t}_{i}$ and ${y}_{i}$, respectively, the following expressions (Freedman et al. 2007) are considered:

- ${SS}_{reg}=\sum\limits_{i=1}^{n}{\left({y}_{i}-\overline{t }\right)}^{2}$: the explained sum of squares.
- ${SS}_{tot}=\sum\limits_{i=1}^{n}{\left({t}_{i}-\overline{t }\right)}^{2}$: this addition is directly related to the variance of the sample.
- ${SS}_{err}=\sum\limits_{i=1}^{n}{\left({t}_{i}-{y}_{i}\right)}^{2}$: the residual sum of squares.$\overline{t }$ is the mean value of the experimental data given by
  20 $$\overline{t }=\frac{1}{n}\sum_{i=1}^{n}{t}_{i}$$
  The coefficient of determination is then defined by the expression (Agresti and Kateri 2021)
  21 $${R}^{2}\equiv 1-\frac{{SS}_{err}}{{SS}_{tot}}$$

The closer the *R*^2^ value to 1, the better the agreement between the observed and foretold values. Additionally, root mean square error (*RMSE*) and mean absolute error (*MAE*) (Agresti and Kateri 2021) are defined as

22 $$RMSE= \sqrt{\frac{1}{n}\sum_{i=1}^{n}{\left({t}_{i}-{y}_{i}\right)}^{2}}$$

23 $$MAE=\frac{1}{n}\sum_{i=1}^{n}\left|{t}_{i}-{y}_{i}\right|$$

The lower the *MAE* and *RMSE* for the model, the closer the actual and predicted values.

Finally, if paired data $\left\{\left({x}_{1},{y}_{i}\right),\dots ,\left({x}_{n},{y}_{n}\right)\right\}$ are considered, the correlation coefficient *r* can be described by (Agresti and Kateri 2021)

24 $$r=\frac{\sum_{i=1}^{n}\left({x}_{i}-\overline{x }\right)\cdot \left({y}_{i}-\overline{y }\right)}{\sqrt{\sum_{i=1}^{n}{\left({x}_{i}-\overline{x }\right)}^{2}\cdot \sqrt{\sum_{i=1}^{n}{\left({y}_{i}-\overline{y }\right)}^{2}}}}$$

where

- *n* is the number of samples;
- ${x}_{i},{y}_{i}$ are the samples; and
- $\overline{x }=\frac{1}{n}{\sum }_{i=1}^{n}{x}_{i}$ is the sample average for variable *x*; and similarly, for $\overline{y }$.

## Results and discussion

Table 1 shows the independent variables of the ABC/SVM, MLP, and M5 models. The dependent variable is the biomass HGP obtained from diverse types of biomass raw samples.

**Table 1:** The independent variables and their means and standard deviations

Input variables	Name of the variable	Mean	Standard deviation
Ash (wt%)	Ash	6.3885	8.5063
Fixed carbon (wt%)	FC	13.719	5.9742
Volatile matter (wt%)	VM	74.574	13.125
Carbon (wt%)	C	47.413	8.3553
Hydrogen (wt%)	H	5.8859	1.0305
Nitrogen (wt%)	N	1.3104	1.0951
Oxygen (wt%)	O	38.465	10.488
Highest treatment temperature (ºC)	HTT	557.55	143.227
Heating rate (ºC/min)	HR	18.774	9.4570
Particle size (mm)	PS	0.9716	1.5244
Inert gas flow rate (mL/min)	FR	130.660	96.651

wt% means weight percentage

The dataset is divided into two sets: 80% is used in the training set and the rest of the data, 20%, is for the testing set. In this sense, the training collection is employed to construct the SVR model. For this purpose, the parameters of the SVR model are calibrated employing the ABC optimiser with a fivefold cross-validation process (Chen et al. 2022). When the optimum parameters have been found, the model is built with the whole training dataset. Then, predictions are obtained with this model for the elements of the testing set. These predictions are compared with the actual values and the goodness of fit of the model evaluated.

In addition, the SVM hyperparameters are *C*, termed *regularisation constant*; *ε*, which defines the width of the insensitive tube and finally, the parameters that are specific to the kernel like *a*, *b*, and *σ*. A common way of tuning the hyperparameters is *grid search* that, as its name indicates, creates a grid of parameters, tries each combination of parameters in the grid, and obtains its goodness of fit. This method is a simple but very time and resource-consuming algorithm and, thus, not very efficient.

**Fig. 4:**
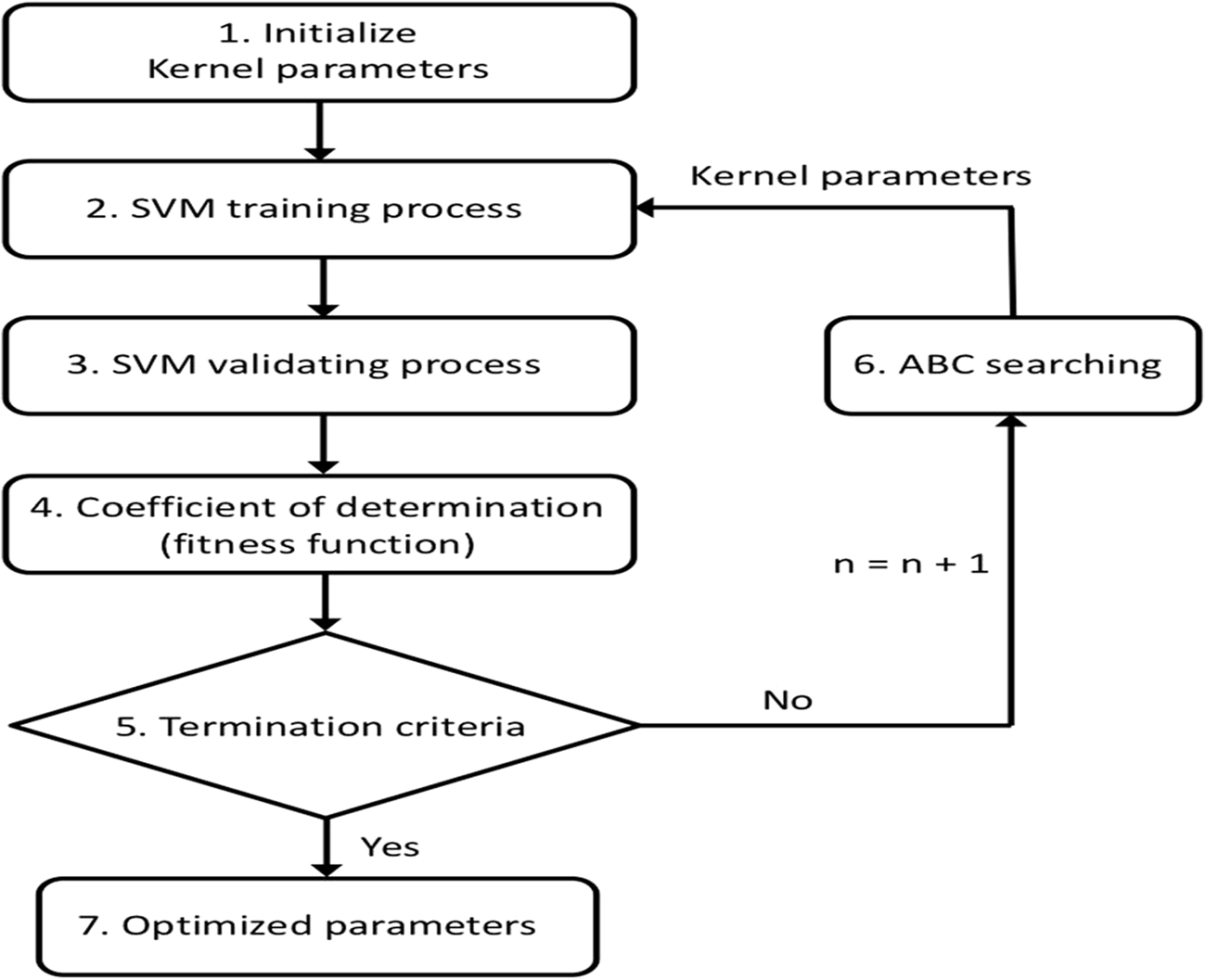
Flow chart for obtaining the ABC/SVM model

In this paper, a more economical method, the ABC optimiser, has been used to obtain the optimal hyperparameters for the SVM model. The employed goodness of fit in the optimisation process was *R*^2^. The flow chart of this procedure is shown in Fig. 4.

In a way, the optimum coefficient of determination (*R*^2^) was also used in the cross-validation methodology (Agresti and Kateri 2021). Fivefold cross-validation allows the selection of the optimal hyperparameters for the ABC/SVM model (Kuhn and Johnson 2018). The data is split into five subsets of similar size in a random way and a set of parameters chosen. Then, a model is constructed with four subsets and checked with the remaining one and a goodness of fit is obtained (Chen et al. 2022). This procedure is repeated five times, employing a distinct subset as testing set each time. The average of the goodness of fit is the final value for the set of parameters that are being used. The ABC algorithm guides the selection of these sets of parameters to try according to their fitness until it decides on a particular group of optimal hyperparameters (Kuhn and Johnson 2018; Chen et al. 2022).

The ABC/SVM models were built using LIBSVM (Chang and Lin 2011) and ABC (Karaboga et al. 2014) for MATLAB. The initial intervals of the hyperparameters for the distinct kernels are indicated in Table 2.

**Table 2:** Initial intervals of the hyperparameters for the different kernels of the ABC/SVM-relied model fitted in this investigation

SVM hyperparameters	Lower limit	Upper limit
*C*	10^–6^	10^4^
*ε*	10^–6^	10^4^
*σ*	10^–6^	10^4^
*a*	0	10^1^
*b*	0	5

The obtained hyperparameters of the SVM calibrated with the metaheuristic ABC optimiser are shown in Table 3.

**Table 3:** Hyperparameters for the SVM approach fitted in this study using the ABC optimiser

Method	Values of optimal hyperparameters
*SVM linear kernel*	Regularisation factor *C* = 1.88 × 10^0^, *ε* = 7.30 × 10^–2^
*SVM polynomial kernel*	Regularisation factor *C* = 2.65 × 10^2^,* ε* = 1.00 × 10^–6^, *σ* = 4.15 × 10^–1^, *a* = 0.5738, *b* = 4.37
*SVM sigmoid kernel*	Regularisation factor *C* = 1.00 × 10^4^,* ε* = 9.23 × 10^–2^,* σ* = 1.20 × 10^–3^, *a* = 0
*SVM RBF kernel*	Regularisation factor *C* = 4.23 × 10^0^,* ε* = 1.31 × 10^–6^,* σ* = 2.42 × 10^0^

For comparison purposes, the MLP neural network and M5 tree approaches have also been used in this paper. MLP accuracy also depends on its parameters (Du and Swamy 2019; Kubat 2021):

- Learning rate (*LR*): This parameter acts in the optimisation of the weights. A small value ensures the convergence, but it can be quite slow. Thus, a balance between convergence and speed must be found.
- Momentum (*m*): It is a coefficient that appears in a term used in the update of the weights.
- Number of hidden layer neurons (*h*): A golden rule is that the hidden layer number of neurons is approximately 2/3 the number of neurons in the input layer size.

Here, the cross-validation method (Agresti and Kateri 2021) with fivefold was employed to determine the coefficient of determination (*R*^2^). The MLP with grid search from WEKA (Hall et al. 2009; Frank et al. 2016) was used. The search space for the MLP parameters is shown in Table 4. The M5 tree model was also obtained with WEKA software.

**Table 4:** Intervals for the MLP parameters in the grid search method

MLP hyperparameters	Lower limit	Upper limit
Learning rate	0	1
Momentum factor	0	1
Number of hidden layers	1	30

The found optimal MLP parameters are indicated in Table 5.

**Table 5:** Optimised parameters in the MLP model

Hyperparameters	Optimal values
Learning rate (*LR*)	0.1
Momentum factor (*m*)	0.7
Number of hidden layers (*h*)	5

**Fig. 5:**
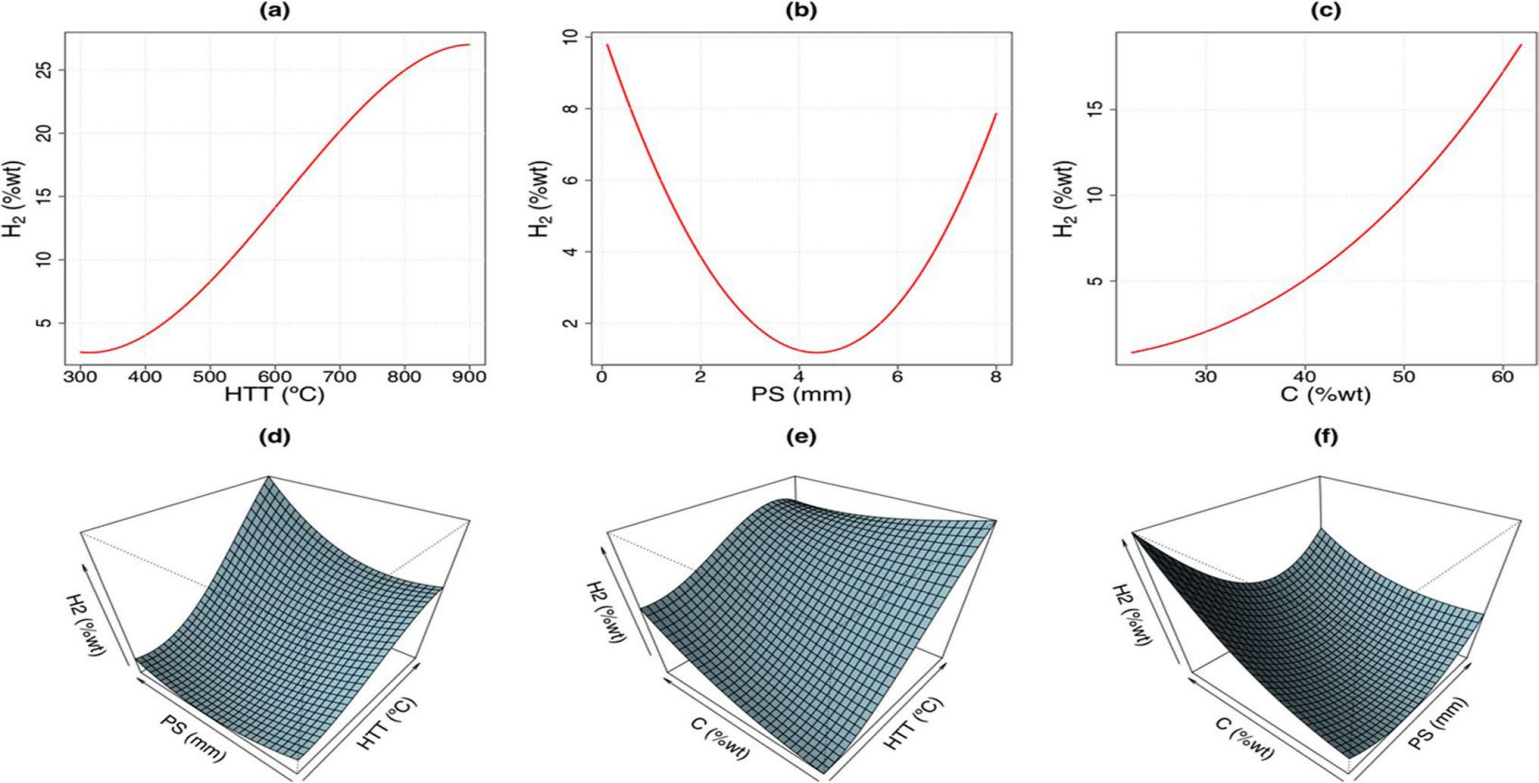
Terms of the first and second order from the ABC/SVM model for the biomass HGP: **a** HTT first-order term; **b** PS term of first order; **c** C term of first order; **d** HTT and PS term of the second order; **e** HTT and C term of second order; and **f** PS and C term of second order

Next, Fig. 5 illustrates the first- and second-order terms for the SVM model using the linear kernel. Figure 5 (a) shows the biomass HGP (*Y*-axis) versus HTT (*X*-axis), while keeping the ten remaining independent variables constant. In fact, from HTT 300 to 900 °C, H_2_ (%wt) is strictly increasing with a maximum of about 27%wt. Similarly, Figs. 5 (b) and (c) illustrate the biomass HGP (*Y*-axis) versus PS (*X*-axis) and versus C (*X*-axis), respectively (also with remaining input variables constant). Figure 5 (b) shows that from PS 1 to 5 mm, H_2_ (%wt) decreases gradually with a minimum of about 1%wt at PS 5 mm. Thereafter, from PS 5 to 8 mm, H_2_ (%wt) increases gradually with a maximum of about 8%wt. Figure 5 (c) indicates that from C (%wt) 20 to 60, H_2_ (%wt) roses gradually and peaked at about 20 at C (%wt) 60. Analogously, Fig. 5 (d) shows the biomass HGP as the dependent variable of the HTT and PS, while the other variables remain constant. Analogously, Figs. 5 (e) and (f) illustrate the biomass HGP as a function of the HTT and C, and PS and C, respectively. Figures 5 (d–f) show the variation of H_2_ (%wt) as a function of each two main input variables, keeping the remaining input variables constant. Therefore, these charts are surfaces in three dimensions. The interpretation is subject to the combination of the one-dimensional variation of the two input variables.

Similarly, Table 6 illustrates the correlation and determination coefficients for the ABC/SVM, SVM without parameter optimisation, MLP, and M5 tree approaches.

**Table 6:** Coefficient of determination (*R*^2^) and correlation coefficient (*r*), mean absolute error (*MAE*), and root mean square error (*RMSE*) for ABC/SVM, SVM, MLP, and M5 tree approach for the test dataset

Model	*RMSE*	*MAE*	*R*^2^	*r*
*ABC/SVM-polynomial*	0.0660	0.0559	**0.9464**	**0.9751**
*SVM-polynomial*	0.2010	1.1753	0.4979	0.8893
*ABC/SVM-RBF*	0.0910	0.0756	0.8971	0.9476
*SVM-RBF*	0.1045	0.0793	0.8643	0.9325
*ABC/SVM-linear*	0.1162	0.0905	0.8320	0.9278
*SVM-linear*	0.1514	0.1301	0.7153	0.9103
*ABC/SVM-sigmoid*	0.1094	0.0806	0.8527	0.9324
*SVM-sigmoid*	0.1175	0.0913	0.8284	0.9197
*M5 model tree*	0.1014	0.0793	0.8722	0.9385
*MLP*	0.1161	0.8853	0.8325	0.9444

Note: The boldfaced entries signify the best fitted model

It is important to consider that 80% of the dataset was employed in the training process, while the testing is done with the remaining 20%. This methodology was built with the training dataset and then used to predict the HGP values of the testing dataset. SVM with the polynomial kernel obtains the best results in the predictions of the biomass HGP with the test set, given that the coefficient of determination *R*^2^ of 0.9464 and a correlation coefficient *r* of 0.9751 are the highest. It took 0.1249 s to obtain the final HGP model with an iMac with a CPU Intel Core i5 @ 3.2 GHz with 8 GB RAM and four cores. Also, the relative importance of the input variables in this model is shown in Table 7.

**Table 7:** Relative relevance ranking for the independent variables implicated in the ABC/SVM-relied approximation for the hydrogen gas foretelling as stated in the associated weights in absolute decreasing order

Input variable	Weight
HTT	0.9638
PS	– 0.3215
C	– 0.3127
Ash	– 0.2521
FC	0.2475
N	0.1580
FR	0.1294
O	0.1109
H	– 0.0704
VM	0.0673
HR	0.0052

Therefore, the most important independent variable in the foretelling of HGP in the ABC/SVM model is HTT, followed by FC, N, FR, and O and in minor contribution VM, HR, H, A, C, and PS.

Several studies have investigated the mechanisms of fuel production through pyrolysis (Cao et al. 2020). Pyrolytic gas is a result of the cracking and decomposition of large molecules present in the raw material during the initial stages of pyrolysis (e.g. CO_2_, H_2_). Hydrogen gas is generated by the decomposition and reforming of aromatic compounds and C–H groups (Hu and Gholizadeh 2019). Moreover, Zanzi et al. (2002) observed that increasing the temperature and reducing the particle size can accelerate the heating rate, leading to a lower char yield. This situation can also promote the cracking of hydrocarbons, resulting in a higher hydrogen content. HTT enhances the production of volatile matter through secondary reactions, leading to the formation of pyrolytic gas, such as decarboxylation, decarbonylation, dehydrogenation, deoxygenation, and cracking (He et al. 2010). These findings suggest that the parameters based on the composition of the biomass (ultimate and proximate analysis) play a critical role in the mathematical model due to the kinetics of the pyrolysis process. Additionally, the analysed FR and PS and the amount of non-inert gas can influence the final compositions and HGP, depending on the chemical equilibrium (Hu and Gholizadeh 2019).

**Fig. 6:**
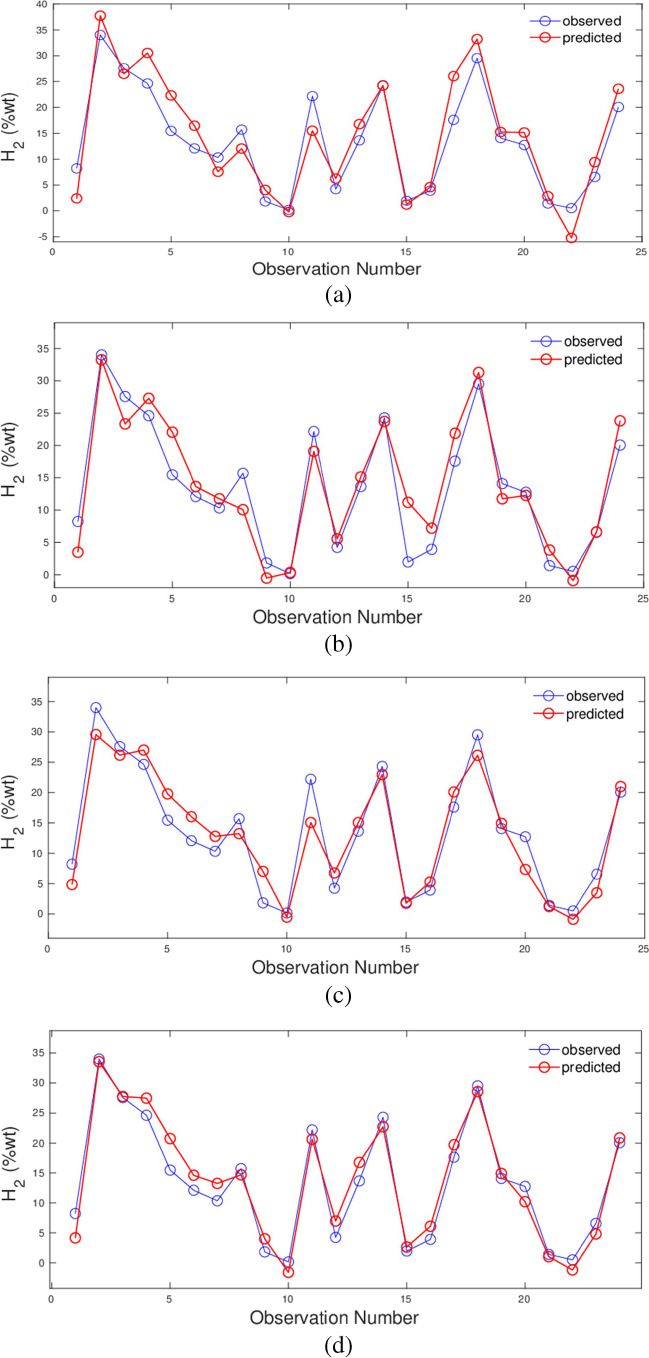
Observed and predicted values of H_2_ production for the test set using **a** MLP model (*R*^2^ = 0.8325); **b** M5 tree model (*R*^2^ = 0.8722); **c** ABC/SVM model with the RBF kernel (*R*^2^ = 0.8971); and ABC/SVM model with the polynomial kernel (*R*.^2^ = 0.9464)

In this investigation, the HGP have been foretold from the independent variables from raw biomass as shown in Fig. 6, utilising the comparison of the observed and foretold HGP examples using the MLP (Fig. 6 (a)), M5 tree (Fig. 6 (b)), ABC/SVM with RBF kernel (Fig. 6 (c)), and ABC/SVM with the polynomial kernel (Fig. 6 (d)) models. The best model is obtained by this fourth model.

To conclude, these techniques can be used with different types of biomass in similar or different bioenergy energy system conversion methods satisfactorily. However, it must be kept in mind the kinds of biomass and experimental environment. Thus, this hybrid ABC/SVM model is an excellent method for the foretelling of HGP. In this sense, one possible direction for future work is to apply this ABC/SVM technique to the production of different combustible gases from the pyrolysis process (e.g. methane and carbon monoxide).

## Conclusion

Different machine learning methods were used to solve this problem and the novel hybrid ABC/SVM approximation employed proved to be an adequate tool to estimate H_2_ production (HGP). The best ABC/SVM approach was obtained with SVR with the polynomial kernel, which got a coefficient of determination of 0.9464 for the testing set. The relative relevance of the independent variables in the prediction of HGP was determined: the variable highest treatment temperature (HTT) proved to be the most direct outstanding in the estimation of HGP. Finally, the HGP values estimated with this approximation concur with the dataset actual values.

## Data Availability

The dataset used and/or analysed during the current study are available from the corresponding author on reasonable request.
